# Exploring Functions and Predictors of Digital Health Engagement Among German Internet Users: Survey Study

**DOI:** 10.2196/44024

**Published:** 2023-06-28

**Authors:** Michael Grimm, Elena Link, Martina Albrecht, Fabian Czerwinski, Eva Baumann, Ralf Suhr

**Affiliations:** 1 Stiftung Gesundheitswissen Berlin Germany; 2 Department of Communication Johannes Gutenberg-University Mainz Mainz Germany; 3 Department of Journalism and Communication Research University of Music, Drama and Media Hanover Hanover Germany

**Keywords:** eHealth, mobile health, digital health engagement, health information seeking, self-monitoring, digital health care, mobile phone

## Abstract

**Background:**

Digital health engagement may serve many support functions, such as providing access to information; checking or evaluating one’s state of health; and tracking, monitoring, or sharing health data. Many digital health engagement behaviors are associated with the potential to reduce inequalities in information and communication. However, initial studies suggest that health inequalities may persist in the digital realm.

**Objective:**

This study aimed to explore the functions of digital health engagement by describing how frequently respective services are used for a range of purposes and how these purposes can be categorized from the users’ perspective. This study also aimed to identify the prerequisites for successfully implementing and using digital health services; therefore, we shed light on the predisposing, enabling, and need factors that may predict digital health engagement for different functions.

**Methods:**

Data were gathered via computer-assisted telephone interviews during the second wave of the German adaption of the Health Information National Trends Survey in 2020 (N=2602). The weighted data set allowed for nationally representative estimates. Our analysis focused on internet users (n=2001). Engagement with digital health services was measured by their reported use for 19 different purposes. Descriptive statistics showed the frequency with which digital health services were used for these purposes. Using a principal component analysis, we identified the underlying functions of these purposes. Using binary logistic regression models, we analyzed which predisposing factors (age and sex), enabling factors (socioeconomic status, health- and information-related self-efficacy, and perceived target efficacy), and need factors (general health status and chronic health condition) can predict the use of the distinguished functions.

**Results:**

Digital health engagement was most commonly linked to acquiring information and less frequently to more active or interactive purposes such as sharing health information with other patients or health professionals. Across all purposes, the principal component analysis identified 2 functions. *Information-related empowerment* comprised items on acquiring health information in various forms, critically assessing one’s state of health, and preventing health problems. In total, 66.62% (1333/2001) of internet users engaged in this behavior. *Health care*–*related organization and communication* included items on patient-provider communication and organizing health care. It was applied by 52.67% (1054/2001) of internet users. Binary logistic regression models showed that the use of both functions was determined by predisposing factors (female and younger age) and certain enabling factors (higher socioeconomic status) and need factors (having a chronic condition).

**Conclusions:**

Although a large share of German internet users engage with digital health services, predictors show that existing health-related disparities prevail in the digital realm. To make use of the potential of digital health services, fostering digital health literacy at different levels, especially in vulnerable groups, is key.

## Introduction

### Functions of Digital Health Engagement

Individuals are increasingly required to engage in maintaining their health, take an active role in their health care, and make informed health-related decisions [1-3]. The era of digital health is characterized by the advancement of information and communication technologies that may enable the development of new solutions for these challenges [4,5]. These technologies range from websites to (prescribed and self-prescribed) apps, tracking devices, and telehealth and medical record systems.

Although these technologies usually have a primary focus, many of them can be used for multiple purposes. This makes it more difficult to state how people engage with their health by simply looking at the respective technology or device. Information on a chronic disease may, for example, be found in health apps, but it can also be gathered via websites [6]. Health data may, for example, be exchanged via electronic health record systems or apps [7].

Thus, to better understand how people digitally engage with their health, it is fruitful to take a *functional perspective* on their use of digital health services, that is, to focus on the purpose of using digital health services instead of examining only which technology or device is used.

Digital health engagement may serve a myriad of support functions, such as accessing information; checking or evaluating an individual’s state of health; promoting self-efficacy, adherence, or changes in health behaviors; tracking, monitoring, or sharing personal health data; or organizing health prevention and health care [8,9].

Existing research on these functions mainly takes 1 of 2 perspectives: the first line of research uses a market perspective. Here, the available digital health services are clustered with respect to the functions that their respective technologies allow. For example, health, provider-comparison, or rating portals that aim to improve health literacy; symptom checkers that aim to analyze health problems and raise awareness; and web-based offices and appointment services that aim to organize and administer health care [8].

The second line of research examines the functions and outcomes of single digital health services developed for and used by specific groups of patients, such as mobile health apps that use built-in smartphone sensors for diagnosis and treatment [10], website-based interventions to support adolescents and young adults with cancer [11], social networking services for people with mental health issues [12], and personal health records to maintain personally reported and clinically sourced data on health care [13,14].

In addition to these 2 lines of research, taking a user’s perspective on the functions of digital health engagement may provide a more holistic perspective, overcoming technology-driven and single-service approaches. However, there are only a limited number of studies [9,15] that describe how frequently digital health services are used for particular purposes and that identify the functions underlying these purposes in a sample of German internet users.

### Potentials of Digital Health Engagement and Their Conditions

A crucial part of the user’s perspective is to examine the characteristics of these users and identify which aspects affect the acceptance and use (or nonuse) of digital health services distinguished by different types of functions. Characterizing users of digital health services is also crucial to identify information and support disparities resulting from different degrees of willingness or abilities to engage with these supportive services [16,17].

On the one hand, the provision of digital health services is associated with the potential to reduce information, communication, and health inequalities [18,19]. These services may increase individuals’ access to health information relevant to health prevention, promotion, and health care; enable affordable access to health care for people with low socioeconomic status (SES); and reduce disparities in health care management between urban and rural populations [4,20].

On the other hand, a narrative review [19] and a study based on data from the US Health Information National Trends Survey (HINTS) [21] suggest that existing health inequalities may persist in the digital realm, as digital health engagement is associated with sociodemographic and socioeconomic factors, as well as abilities such as digital health literacy.

Therefore, the increasing availability of digital health services is only 1 component that is necessary so digital solutions can support the active engagement of individuals in health care. Beyond the advancements in technologies, an individual’s willingness and abilities, which manifest in differences in user behavior, are crucial to benefit from digital health services and to participate in medical progress [19]. Accounting for an individual’s willingness and abilities has become even more important in the digital era, as digital health engagement requires a higher level of involvement and personal responsibility of the users.

Against this background, we consider predictors proven relevant to explain health information–seeking behaviors (HISBs) or the use of health applications [19,22,23] to learn more about the users of digital health services, distinguished by the purpose of their use and who is reached or remains unreached by the various types of digital health services.

Known *predisposing factors* for the use of health applications are sociodemographic and socioeconomic characteristics such as age and sex [4,5,15,20,24].

SES is also an *enabling factor* in the use of digital health services [15]. In line with the increased importance of digital health engagement abilities, different types of efficacy assessments are additional enabling factors that may characterize users. Efficacy assessments describe an individual’s self- and target-related perception of possessing the ability to perform a task or enact a behavior, such as using digital health services for various purposes [25,26]. These assessments are integral parts of models that explain information or web-based support seeking [26-29]. Focusing on self-related efficacy assessments, we consider health-related and information-related self-efficacies to examine whether more pronounced efficacies to take good care of one’s health and complete communication tasks such as information and web-based support seeking [26] are associated with different purposes of the use of digital health services. Focusing on the target efficacy regarding digital health engagement, we considered whether the internet is perceived as a trustworthy source for health purposes.

Other dimensions relevant to characterizing users of various digital health services are *need factors* [15] such as their subjective health status and whether they are affected by chronic disease. We assume that both determine the personal relevance of using digital health services and the specific purpose of use.

In summary, predisposing, enabling, and need factors should be considered to increase the understanding of the respective patterns of digital health engagement for various purposes.

### Objectives

Digitally engaging with one’s health is common in many Western countries. In Germany, several acts passed by the Ministry of Health have recently promoted digital transformation of the health care system [30,31]. Digital health applications that can be prescribed by physicians, for example, now offer a new form of digital health care [32].

However, there are also challenges to digital health engagement resulting from the country-specific situation in Germany: data security and privacy issues are of particular importance in Germany and sometimes conflict with the aims of digital health services [31]. In addition, as the availability of digital health services increases, users need to have adequate digital health literacy to make use of these innovations [30,33]. However, recent studies show that the digital health literacy of the German population is rather limited [33].

Thus, Germany is a particularly interesting case to look at when we research digital health engagement. Until recently, representative data on how digital health services were used in Germany were limited [34]. In the United States, the HINTS, initiated by the National Cancer Institute since 2003, regularly provides cross-sectional data on the access to, need for, and use of health information and allows the analysis of trend over time [35]. To establish a comparable, nationally representative trend study, the survey was adapted in Germany in 2018 [36]. Subsequently, 2 waves of data collection have been conducted.

This study aimed to complement the limited evidence on the use of digital health services for various functions by analyzing data from the second wave of HINTS Germany.

First, we aimed to explore the use of digital health services by describing how frequently people engage with them for a range of purposes (refer to research question [RQ] 1) and by categorizing different functions of digital health engagement (refer to RQ2).

- RQ1: How frequently do German internet users engage with digital health services for different purposes?
- RQ2: Which functions of digital health services can be distinguished, and how often are they used?

Second, we aimed to identify the prerequisites for the successful implementation and use of digital health services by shedding light on predisposing, enabling, and need factors associated with digital health engagement for different types of functions (refer to RQ3). Although current research on predictors of HISB indicates correlates of digital health engagement for single digital health services and may allow us to derive hypotheses for each factor, we opt for a more open RQ because the distinguished functions of digital health services are not developed in a theory-driven manner but by using an explorative empirical approach.

- RQ3: Which predisposing, enabling, and need factors are associated with the use of the distinguished functions of digital health engagement?

## Methods

### Data Collection and Sample

To answer our RQs, we analyzed data from the second wave of the German HINTS. As a franchise trademark of HINTS United States, HINTS Germany started in 2018 as a cooperative project between the Stiftung Gesundheitswissen and the Hanover Center for Health Communication (for a further description refer to the study by Link et al [36]). The main goal of the project was to establish a nationally representative survey that collected data on several topics of health information seeking, with a close methodological analogy to the HINTS United States original.

A representative sample of German residents was recruited using a 2-stage sampling approach applied within a computer-assisted telephone interview dual-frame survey (40% mobile and 60% landline). Trained interviewers posed questions and recorded the interviewees’ responses. The sampling of the German respondents was implemented on the basis of the reference sampling system for representative studies in Germany [36]. Although users in the mobile frame were directly chosen as respondents, among the landline sample frame, the Kish-grid was used to randomly select 1 of the eligible adult household members as the interviewee.

The second wave of HINTS Germany was conducted from May 2020 to August 2020. In total, 2602 respondents participated in the second wave, the median interview length was 32 minutes, and the response rate (according to the type 3 calculation formula of the American Association of Public Opinion Research [37]) was 19.4% (refer to the study by Finney et al [35] for a comparison with HINTS United States). The mean age was 48.2 (SD 17) years, and 50.12% (1304/2602) of the sample were female.

Data were weighted by federal statistical information on the distribution of sex, age, level of education, marital status, and county of residence across the entire German population. Therefore, the data allowed for the calculation of nationally representative estimates. In this paper, we focus on internet users only. Therefore, the findings are representative of German internet users (2001/2602, 76.9%). As can be seen in Table 1, internet users tend to be somewhat younger and have a slightly higher SES compared with the total sample.

**Table 1:** Descriptive statistics of the predisposing, enabling, and need factors.

Variable			Total sample (N=2602)				Internet users only (n=2001)		
			Respondents, n (%)		Value, mean (SD)		Respondents, n (%)		Value, mean (SD)
Age (years)			N/A^a^		48.2 (17)		N/A		44.8 (15.9)
**Sex**									
	Female	1304 (50.1)		N/A		989 (49.4)		N/A	
	Male	1298 (49.9)		N/A		1011 (50.6)		N/A	
**Socioeconomic status (total: n=2483 and internet users only: n=1921)**									
	Low	673 (27.1)		N/A		407 (20.4)		N/A	
	Medium	1164 (46.9)		N/A		932 (46.6)		N/A	
	High	646 (26)		N/A		582 (29.1)		N/A	
**General health status (total: n=2569 and internet users only: n=1970)**									
	Very bad, bad, or moderate	701 (27.3)		N/A		463 (23.5)		N/A	
	Good or very good	1868 (72.7)		N/A		1507 (76.5)		N/A	
Health-related self-efficacy			N/A		4.08 (0.84)		N/A		4.13 (0.79)
Information-related self-efficacy			N/A		3.55 (0.92)		N/A		3.57 (0.92)
Perceived target efficacy			N/A		2.67 (1.06)		N/A		2.80 (0.98)
**Chronic disease (internet users only: n=2000)**									
	Yes	1447 (55.6)		N/A		1062 (53.1)		N/A	
	No	1155 (44.4)		N/A		938 (46.9)		N/A	

^a^N/A: not applicable.

### Ethical Considerations, Informed Consent, and Participation

The applied type of data collection, according to German standards, was considered exempt from ethics approval. Data collection was conducted following the Declaration of Helsinki and the standards of the German National Communication Association (§2 Rechte von Untersuchungspersonen [Rights of study participants] of the Ethik-Kodex) and the German Research Foundation. All participants were informed about the scientific purpose of the research, were asked for informed consent at the beginning of the survey, and were advised of their right to cancel participation.

### Measures

#### Overview

For the second wave of HINTS Germany, several changes were made to the instrument of the first wave of HINTS Germany, which was developed based on the instrument of HINTS United States 5, cycle 1 (refer to the study by Link et al [36] for further information on the adaption of HINTS United States to Germany). The German instrument for wave 2 was supplemented by the HINTS 5, cycle 3 instrument. Relevant adjustments are noted in the description of the measures used.

#### Purposes of Digital Health Engagement

For the second wave of HINTS Germany, individual measurements of the validated first wave questionnaire [38] focusing on the purposes of digital health engagement were compiled into an overarching item set to allow for a more consistent data collection. The item set was then compared with a technology-oriented typology of digital health services and their purposes [8] to strengthen the conceptual underpin and check for thoroughness. For those 3 purposes that were included in the typology but were not yet part of the measurements of the HINTS questionnaire, additional items were compiled and included in the item set. We put a particular focus on the extended item set in the pretest with 50 completed interviews to ensure the quality of the instrument for the second wave [39]. Adaptions to the instrument can also be found in the respective methodology report [40].

With the item set, respondents of HINTS Germany were asked whether they had used digital health services for the resulting 19 different purposes in the last 12 months (eg, “searching for health information for myself’” or “making appointments with health professionals”). In contrast to the US instrument asking only yes or no, the German measurement distinguishes whether the respondents already use digital services, have not yet used a respective service but intend to do so in the future, or none of the above. This adoption was made because in Germany, the implementation of digital services is less developed than in other Western countries.

#### Predisposing, Enabling, and Need Factors of Digital Health Engagement

In addition to sex and age, which are predisposing factors, the SES of respondents was included as an enabling factor (Table 1). It was calculated as a function of the weighted household income and the level of education, indicating either a low, medium, or high SES. Other enabling factors considered were 2 types of self-efficacy assessments: health- and information-related self-efficacy. They were measured in line with the HINTS United States and HINTS Germany measurements, using 1 item per efficacy assessment. Respondents’ health-related self-efficacy was assessed using an item asking for their self-rated confidence in taking good care of their health. Information-related self-efficacy was assessed by asking for the participants’ self-rated confidence in their ability to obtain information about health or medical topics when needed. Both statements were measured using 5-point Likert-type scales ranging from 1 (“not confident at all”) to 5 (“completely confident”). Perceived target efficacy was evaluated with a single item asking about the extent to which the internet is perceived as a trustworthy source of health information. Responses ranged from 0 (“not at all”) to 4 (“a lot”).

Regarding need factors for use, respondents’ general health status was assessed with an item asking them to rate their health on a 5-point Likert-type scale ranging from 1 (“very good”) to 5 (“very bad”). Owing to the strong skewness of this measure, a dummy was built to compare respondents stating good or very good health with those rating their health as very bad, bad, or moderate. Furthermore, we combined the responses on 6 explicitly assessed chronic conditions (diabetes, hypertension, heart disease, chronic obstructive pulmonary disease, arthritis or rheumatism, and depression or anxiety disorder) into 1 single dummy to indicate whether respondents were affected by at least 1 chronic health condition.

### Data Analysis Procedures

To examine how frequently German internet users digitally engage with their health with respect to different purposes (RQ1), we provide descriptive statistics for the 19 items included in the survey.

To analyze which functions of digital health engagement can be distinguished (RQ2), we conducted a principal component analysis (PCA) [41] of these variables. The items were recoded into dummies, contrasting the “recently used” answers with the 2 other categories (“no” and “maybe in the future”). On the basis of the tetrachoric correlation matrix of these dummies, rotation techniques and best practice recommendations were applied to search for a consistent solution.

Next, we aimed to analyze which predisposing, enabling, and need factors can predict the use of the distinguished functions (RQ3). To answer this question, we calculated 2 binary logistic regression models [42]. As dependent variables, we used the engagement with digital health services (at least 1 purpose associated with it) for each extracted component of the PCA. As independent variables, we considered respondents’ predisposing (age and sex), enabling (SES and various forms of self-efficacy), and need (health status and chronic disease) factors. Across these multivariate analyses, we applied the jackknife replicate weights included in the data set to ensure proper SEs [43], using Stata (version 15.1; StataCorp) [44]. Owing to the complex survey structure of the data and the corresponding data analysis, no conventional measure of pseudo-*R*² was obtained. Missing values were deleted listwise, and the type I error rate was set to 0.05 across all analyses.

## Results

### Frequency of Use for Various Purposes of Digital Health Engagement

Regarding our RQ1 on how frequently German internet users engage with digital health services for different purposes (Table 2), the findings showed that most participants used digital health services in the past 12 months “to look for a health care provider” (979/1997, 49.02%), followed by “to look for health or medical information for yourself” (925/1990, 46.48%). Approximately, 1 in 3 respondents reported using digital health services “to look for health or medical information for someone else” (660/1990, 33.17%) and “to make appointments with a health care provider” (656/1995, 32.88%). In contrast, the purposes “to share health information or talk with people having a similar health or medical issue” (280/1993, 14.05%) and “to share health information from either an electronic monitoring device or smartphone with a health professional” (203/1995, 10.17%) had the lowest rates of use in the last 12 months. In terms of future purposes of use, respondents attributed the highest potential to the purposes “to look up test results” (785/1993, 39.39%) and “to track health care charges and costs” (709/1993, 35.57%).

**Table 2:** Purposes of digital health engagement^a^.

Purpose	Use, n (%)		
	Yes, I used it in the last 12 months	Not yet, but maybe in the future	I did not use it, nor do I intend to in the future
...to look for a health care provider (n=1997)	979 (49)	564 (28.2)	455 (22.8)
...to look for health or medical information for yourself (n=1990)	925 (46.5)	496 (24.9)	570 (28.6)
...to look for health or medical information for someone else (n=1990)	660 (33.1)	547 (27.4)	786 (39.4)
...to make appointments with a health care provider (n=1995)	656 (32.9)	682 (34.2)	658 (33)
…to watch a health-related video (n=1996)	608 (30.5)	622 (31.2)	764 (38.3)
…to buy medicine or vitamins online (n=1998)	586 (29.3)	341 (17.1)	1071 (53.6)
…to prevent health problems or to treat them^b^ (n=1994)	550 (27.6)	592 (29.7)	853 (42.8)
...to help you decide how to treat an illness or condition (n=1996)	548 (27.5)	631 (31.6)	816 (40.9)
...to find out whether you have a health problem^b^ (n=1998)	530 (26.5)	479 (24)	989 (49.5)
…to track your progress on a health-related goal such as quitting smoking, losing weight, or increasing physical activity (n=1985)	504 (25.4)	511 (25.7)	970 (48.9)
...to fill out forms or paperwork related to your health care (n=1995)	483 (24.2)	658 (33)	854 (42.8)
...to communicate with a physician, a physician’s office, or other health professionals (via email, WhatsApp, etc) (n=1994)	461 (23.1)	682 (34.2)	852 (42.7)
...to learn how to get along in the health care system better^b^ (n=1990)	396 (19.9)	697 (35)	897 (45.1)
...to help you in discussions with your health care provider (n=1990)	395 (19.9)	656 (33)	939 (47.2)
...to continuously record and evaluate information about your health (n=1994)	354 (17.8)	547 (27.4)	1093 (54.8)
...to look up test results (n=1993)	347 (17.4)	785 (39.4)	860 (43.2)
...to track health care charges and costs (n=1993)	338 (17)	709 (35.6)	946 (47.5)
...to share health information or talk with people having similar health or medical issues (n=1993)	280 (14.1)	571 (28.6)	1142 (57.3)
...to share health information from either an electronic monitoring device or smartphone with a health professional? This includes data on your blood pressure or your heart rate, for example (n=1995)	203 (10.2)	704 (35.3)	1089 (54.6)

^a^Question: “Meanwhile, there is a whole range of digital health services you can use on a computer, tablet, or smartphone. The reasons for people to engage with these services can vary. I will now read out possible reasons and you please tell me, whether you have used an offer for this reason in the last 12 months or whether you can imagine using it in the future.”

^b^Items added after comparing the US Health Information National Trends Survey instrument with the classification [8].

### Distinguishing Different Functions of Digital Health Engagement

When distinguishing different functions of digital health engagement (see RQ2), the data showed a sufficiently high measure of sampling adequacy (Kaiser-Meyer-Olkin=0.94), and the first run of the PCA resulted in a solution with 3 components with an eigenvalue >1 and an explained variance of 57.7%. However, a couple of items exhibited problems with double loadings on at least 2 factors; therefore, the solution was not unique. We excluded items where the difference between loadings was <0.2 and reran the analysis. With the remaining 12 items, the PCA resulted in a 2-factor solution with a clear structure of loadings. To enhance the interpretability of the solution while relaxing the assumption that the components are independent of each other, an oblique rotation technique (oblimin) was applied. The resulting solution consisted of 2 components, explaining 89.4% of the total variance. The first component comprised 7 items, and the second component comprised 5 items. The loadings are shown in Table 3 and range from 0.597 to 0.834. Most residuals were <0.05, thus posing no problems to the solution [45].

Regarding the items included, the first component was labeled *information-focused empowerment*, which represented acquiring health information in various forms, critically assessing one’s state of health, and preventing health problems as core purposes. The second component was called *health care*–*related organization and communication*, where patient-provider communication and organizing health care were the most important aspects.

The results for the intensity of use of both components are presented in Table 4. A total of 66.62% (1331/1998) of German internet users engaged with digital health services for at least 1 purpose associated with *information-focused empowerment*, whereas 52.7% (1053/1998) of German internet users used at least 1 purpose related to *health care*–*related organization and communication*.

Referring to the first function, *information-focused empowerment*, 14.01% (280/1998) of participants engaged with digital health services for 1 purpose during the last year, 12.41% (248/1998) for 2 purposes, and 15.52% (310/1998) for 3 purposes. Nearly one in four (494/1998, 24.7%) participants stated that they had used digital health services for >4 purposes linked to the first component.

Regarding the second component, *health care*–*related organization and communication*, 22.07% (441/1998) of participants reported having engaged with digital health services for 1 purpose and 14.91% (298/1998) for 2 purposes associated with the component; 15.51% (310/1998) of participants stated that they used digital health services for 3 to 5 purposes linked to the second component.

**Table 3:** Factor loadings (oblimin-rotated solution; n=1998).

Purpose	Information-focused empowerment, factor loading	Health care–related organization and communication, factor loading
...to find out whether you have a health problem	0.834	—^a^
...to help you decide how to treat an illness or condition	0.809	—
...to look for health or medical information for yourself	0.807	—
...to look for health or medical information for someone else	0.788	—
...to prevent health problems or to treat them	0.764	—
...to watch a health-related video	0.749	—
...to help you in discussions with your health care provider	0.597	—
...to make appointments with a health care provider	—	0.822
... to communicate with a physician, a physician’s office, or other health professionals (via email, WhatsApp, etc)	—	0.813
...to fill out forms or paperwork related to your health care	—	0.762
...to track health care charges and costs	—	0.642
...to share health information from either an electronic monitoring device or smartphone with a health professional? This includes data on your blood pressure or your heart rate, for example	—	0.631

^a^Factor loadings <0.3.

**Table 4:** Intensity of use among the 2 components (n=1998).

Number of purposes for which digital health services were used during the last 12 months	Information-focused empowerment, n (%)	Health care–related organization and communication, n (%)
0	667 (33.4)	945 (47.3)
1	280 (14)	441 (22.1)
2	248 (12.4)	298 (14.9)
3	310 (15.5)	182 (9.1)
4	182 (9.1)	98 (4.9)
5	132 (6.6)	34 (1.7)
6	120 (6)	—^a^
7	60 (3)	—

^a^No respondents used digital health services for more than 5 purposes associated with health care–related organization and communication.

### Predictors to Use Different Functions of Digital Health Engagement

Regarding RQ3, the multivariate analysis of the predictors of digital health engagement for the 2 functions resulted in findings depicted in Table 5. The estimates of the regression models are displayed as odds ratios (ORs) and relate to the overall frequency of use (whether digital health services were used for at least 1 purpose in the last 12 months) observed in the survey.

**Table 5:** Results of the logistic regression models on the use of the 2 functions^a^.

Variable				Information-focused empowerment (n=1860)					Health care–related organization and communication (n=1866)		
				OR^b^ (95% CI)		*P* value		OR (95% CI)			*P* value
**Predisposing factors**											
	Sex: female (reference: male)			1.90 (1.36-2.65)		<.001		1.47 (1.14-1.89)			.004
	**Age (years; reference: 18-39)**										
		40-59	0.61 (0.42-0.88)		.01		0.82 (0.54-1.24)			.33	
		60-79	0.62 (0.43-0.90)		.01		0.78 (0.54-1.12)			.17	
**Enabling factors**											
	**SES^c^ (reference: low SES)**										
		Medium SES	1.25 (0.79-1.98)		.32		1.31 (0.82-2.09)			.25	
		High SES	1.98 (1.31-2.98)		.002		2.31 (1.52-3.52)			<.001	
	Information-related self-efficacy			1.09 (0.90-1.31)		.37		1.04 (0.87-1.24)			.66
	Health-related self-efficacy			0.87 (0.69-1.10)		.23		1.01 (0.81-1.26)			.91
	Trust in web-based health information			1.44 (1.25-1.66)		<.001		1.15 (0.99-1.33)			.06
**Need factors**											
	Health status: good or excellent (reference: moderate, bad, or very bad)			0.77 (0.54-1.11)		.16		1.03 (0.65-1.61)			.91
	Chronic disease: yes (reference: no)			1.47 (1.00-2.14)		.049		1.39 (1.05-1.85)			.02

^a^Missing cases were deleted listwise.

^b^OR: odds ratio.

^c^SES: socioeconomic status.

Regarding the function *information-focused empowerment*, all predisposing factors showed significant effects: female respondents were expected to have a 1.9-fold higher chance of engaging with digital health services for at least 1 associated purpose than male respondents (95% CI 1.36-2.65; *P*<.001). Compared with the reference group (aged 18-39 years), digital health engagement reported by respondents from the highest age group (60-79 years) decreased by a factor of 0.62 (95% CI 0.43-0.90; *P*=.01).

Regarding the enabling factors, the findings showed that a high SES was associated with an increase in digital health services use for information-focused empowerment by a factor of 1.98 (95% CI 1.31-2.98; *P*=.002) when compared with the reference group with a low SES. A 1-point increase in respondents’ trust in health information obtained on the internet was accompanied by 1.44-times higher odds of having used digital health services during the last 12 months (95% CI 1.25-1.66; *P*<.001). However, the respondents’ information-related (OR 1.09, 95% CI 0.90-1.31; *P*=.37) and health-related self-efficacy (OR 0.87, 95% CI 0.69-1.10; *P*=.23) measures were not associated with the use of digital health services for information-focused empowerment.

For the need factors, the findings indicated that good or excellent self-rated health of the respondents did not significantly decrease the use of digital health services (OR 0.77, 95% CI 0.54-1.11; *P*=.16). Respondents with at least 1 chronic disease showed significantly increased odds of expected use (OR 1.47, 95% CI 1.00-2.14; *P*=.04).

The results concerning the use of digital health services for *health care*–*related organization and communication* showed 3 significant findings. First, female respondents showed a 1.47-fold higher chance of engaging with digital health services for at least 1 respective purpose than male respondents (95% CI 1.14-1.89; *P*=.004). Second, a high SES increased the odds of having used a digital health service for a respective purpose by a factor of 2.31 (95% CI 1.52-3.52; *P*≤.001) compared with respondents with a low SES. Third, those affected by at least 1 chronic disease showed a significant increase of 39% (OR 1.39, 95% CI 1.05-1.85; *P*=.02).

In sum, being female, being younger, and having a high SES in combination with the presence of chronic health conditions significantly increased the odds of engaging with digital health services for health care–related organization and communication in the last 12 months.

## Discussion

### Principal Findings

#### Purposes of Digital Health Engagement

Digital health services provide individuals with a broad spectrum of functions that may support their engagement, participation, and management of health prevention and health care [8,9]. We aimed to stress the users’ perspective and provide an overview of the purposes and functions of the use of digital health services among German internet users [15]. RQ1 addressed how frequently they engaged with digital health services for specific purposes. Our findings showed that digital health services were most frequently used to access and acquire information or support [8], which is a known pattern of web-based health information seeking in general [46,47]. In contrast, purposes that require more activity, participation, or interaction of individuals such as sharing health information with similar others or monitoring health features [9] were the least used by respondents. This may be related to the fact that these are more likely to gain importance for specific patient groups when certain need factors such as an acute or chronic disease are prevalent [48]. Furthermore, monitoring and tracking functions are newer and, therefore, naturally still are in the earlier steps of adaption.

#### Functions of Digital Health Engagement

Starting with individual purposes, our RQ2 aimed to identify the types of purposes of digital health engagement. We extended the existing classifications of available services from a market perspective [8] using a user-based perspective. By statistically aggregating the use for specific purposes, 2 dominant functions were identified: the first function was called *information-related empowerment*, whereas the second function was labeled *health care*–*related organization and communication*.

The function *information-related empowerment* comprised purposes that aim at fostering individuals’ empowerment to participate in health prevention and health care such as to acquire available knowledge on a health or illness issue, enable individuals to act in a self-determined way, and take actions within and outside the health care system [19]. This function may be addressed by digital health services such as health information portals designed for the general public as well as for specific patient groups. Comparing this focus with market-based classifications [8], this component might be the user counterpart for the type called “improve health literacy” comprising digital health services that provide information regarding health or illness-related issues.

The function *health care*–*related organization and communication* encompassed purposes related to instrumental support for appointments with health professionals (such as making an appointment via the appointment systems of providers), helping to navigate the health care system, and empowering patients to be prepared for communication with health professionals. In addition, this component includes monitoring, tracking, and sharing health and illness records via smartphones or fitness apps. Compared with market-based classifications, this function refers to aspects of the types of “organization and administration” describing the process management in health care and “health and illness history documentation” [8].

#### Predicting Digital Health Engagement for Various Functions

Considering the predictors of the use of the identified functions of digital health engagement (RQ3), our findings revealed that people were more likely to use both functions when they were female, were younger, had a high SES, or had a chronic disease. Thus, the use was determined by predisposing factors and also by certain enabling and need factors [15], which is in line with previous findings [4,5,20,24]. In particular, for the function *information-related empowerment*, trust in web-based health information as a type of target efficacy [26] served as a promoting factor. In contrast, it is rather surprising that health- and information-related self-efficacy, which are established predictors of HISB [22,23], were not associated with the use of digital health services for these 2 functions. One reason for the lack of an association may be that the influence of self-efficacy depends on an individual’s health status, as suggested by recent studies [48]. Although self-efficacy is not a promoting factor for use, the need factor of being affected by a chronic disease and enabling factors such as SES provide support for extant findings that the existing disparities seem to continue into the digital realm [18,19]. We observed that both functions were less likely to be used by groups of people who were already affected by health-related disparities, such as older people or people with a lower SES. To fulfill the promise that digital health services may help alleviate health inequalities, it is crucial to assist these groups in using the respective functions of digital health services in a meaningful way. Effective measures should be undertaken to facilitate digital health engagement by all relevant groups and to thereby reduce existing disparities.

### Limitations and Future Research Tasks

Although this study identifies distinct functions of digital health services, predictors, and barriers regarding their use, thereby extending existing research, the limitations of our study need to be considered. First, a key limitation of this study is that the data were cross-sectional and did not allow for causal inference. Second, the use of single-item measures for many of our independent variables is also a methodological shortcoming, so the associations we found or did not find for RQ3 may be impaired by measurement issues. Further research should use multi-item measures to examine in more detail the relationship between various types of self-efficacy and digital health engagement. Third, modifications in the questionnaire between the extensively validated first wave instrument and the second wave instrument were scrutinized and optimized through a pretest with 50 completed interviews. Learnings from the compilation of single items into an overarching item set and the addition of items may be considered in the advancement of the HINTS questionnaires in German and other languages. Fourth, although the identified functions account for a large share of the purposes of digital health engagement, we must critically reflect on the additional purposes for which digital health services may be used. Several of them were collected with respective questionnaire items but had to be excluded during data analysis because of double loadings. Thus, information-related empowerment and health care–related organization and communication are key functions, but market-based approaches [8] suggest that digital health services potentially serve a broader set of functions. Further research could use our classification as a starting point or map the market perspective to provide deeper insights into the broad spectrum of digital health engagement purposes. Fifth, the data of our sample refer exclusively to Germany, so our findings need to be investigated in other countries as well. Our findings should also be interpreted with respect to the contextual influences during the second wave of HINTS Germany, which was fielded from May 2020 to August 2020. The COVID-19 pandemic might have abetted the use of digital health services; therefore, the corresponding results are context sensitive and must be proven in future surveys.

### Practical Implications

#### Overview

Digital health services have found their way into everyday life in Germany; a large share of German internet users engage with them for information-related empowerment and health care–related organization and communication. This acceptance and adaption allow for the promising advancement of respective services and continued digital supplementation of health care.

However, predictors show that existing health-related disparities, for example, with respect to age or SES, prevail in the digital realm. Although these predisposing and enabling factors cannot or can hardly be changed, it is essential to assist vulnerable groups to make use of the potential that digital health services may provide. One of the main obstacles regarding this may be a lack of digital health literacy [49], which encompasses a set of skills that may be key in the context of future health care. Fostering an individual’s ability to find, evaluate, and apply health information may increase trust and affinity and reduce concerns. Structural measures may help create environments that better enable health-literate behavior.

Therefore, we see the following practical implications for service providers, actors in the health care system, and political stakeholders to improve digital health literacy and, thereby, reduce health-related disparities:

#### Applying Setting-Oriented Approaches

It is crucial to foster digital health literacy across groups of people from different backgrounds such as SES. Setting-oriented approaches are a promising means to achieve this goal and help alleviate health inequalities. They situate interventions in the context of people’s everyday lives [50], thereby allowing to reach out to people with different predisposing, enabling, and need factors and particularly to vulnerable groups that can hardly be reached otherwise. Considering specific settings, 2 exemplary contexts might be fruitful. First, the school context allows health literacy to be fostered in the early stages of life across diverse groups of people. The first findings indicate that electronic learning courses on health literacy may have a positive impact on self-reported (digital) health literacy and knowledge acquisition [51]. In addition, integrating digital health literacy into school curricula could address this issue at the structural level. Second, the general practitioner’s office functions as an anchor for people navigating the health care system and constitutes a promising context for health promotion and illness prevention [52]. Here, certain need factors such as chronic diseases are frequently prevalent and make the use of digital health services more likely. Both settings allow not only to reach people from various backgrounds but also to address the settings themselves by improving structures and processes as well as the capacities of actors within the setting.

#### Improving and Adapting Digital Health Services to User Behavior

The 2 identified main functions for users (*information-related empowerment* and *health care*–*related organization and communication*) show which aspects deserve particular attention in advancing digital health services. For instance, information-related empowerment may be addressed by health information portals that provide knowledge on health and illness issues, show how to navigate the health care system and how to communicate with providers, and support abilities and motivation to act according to one’s preferences. Furthermore, as trust in web-based health information serves as a promoting factor for information-related empowerment, service providers may generate evidence on the usefulness of particular services and implement standards for “good” information, such as the Guideline Evidence-based Health Information [53], to address uncertainty about the quality of information.

## Abbreviations

HINTS: Health Information National Trends Survey
HISB: health information–seeking behavior
OR: odds ratio
PCA: principal component analysis
RQ: research question
SES: socioeconomic status

## Data Availability

The data set analyzed during this study is available on reasonable request via open access [54].
